# Mycorrhizal symbiosis primes the accumulation of antiherbivore compounds and enhances herbivore mortality in tomato

**DOI:** 10.1093/jxb/erab171

**Published:** 2021-04-22

**Authors:** Javier Rivero, Javier Lidoy, Ángel Llopis-Giménez, Salvador Herrero, Víctor Flors, María J Pozo

**Affiliations:** 1 Department of Soil Microbiology and Symbiotic Systems, Estación Experimental del Zaidín, Consejo Superior de Investigaciones Científicas (CSIC), Granada, Spain; 2 Department of Genetics and Institut Universitari en Biotecnologia i Biomedicina (BIOTECMED), Universitat de València, Burjassot, Spain; 3 Metabolic Integration and Cell Signaling Laboratory, Plant Physiology Section, Unidad Asociada al Consejo Superior de Investigaciones Científicas (EEZ-CSIC)-Department of Ciencias Agrarias y del Medio Natural, Universitat Jaume I, Castellón, Spain; 4 MPI for Molecular Plant Physiology, Germany

**Keywords:** Arbuscular mycorrhiza, defence priming, herbivory, metabolomics, mycorrhiza induced resistance, *Spodoptera exigua*

## Abstract

Plant association with arbuscular mycorrhizal fungi (AMF) can increase their ability to overcome multiple stresses, but their impact on plant interactions with herbivorous insects is controversial. Here we show higher mortality of the leaf-chewer *Spodoptera exigua* when fed on tomato plants colonized by the AMF *Funneliformis mosseae*, evidencing mycorrhiza-induced resistance. In search of the underlying mechanisms, an untargeted metabolomic analysis through ultra-performance liquid chromatography tandem mass spectrometry (UPLC-MS) was performed. The results showed that mycorrhizal symbiosis had a very limited impact on the leaf metabolome in the absence of stress, but significantly modulated the response to herbivory in the damaged area. A cluster of over accumulated metabolites was identified in those leaflets damaged by *S. exigua* feeding in mycorrhizal plants, while unwounded distal leaflets responded similar to those from non-mycorrhizal plants. These primed-compounds were mostly related to alkaloids, fatty acid derivatives and phenylpropanoid-polyamine conjugates. The deleterious effect on larval survival of some of these compounds, including the alkaloid physostigmine, the fatty acid derivatives 4-oxododecanedioic acid and azelaic acid, was confirmed. Thus, our results evidence the impact of AMF on metabolic reprograming upon herbivory that leads to a primed accumulation of defensive compounds.

## Teaser

Mycorrhizal symbiosis primes the accumulation of several antiherbivore compounds in response to *Spodoptera exigua* attack, increasing larval mortality.

## Introduction

Plants are highly dynamic systems that are able to react to, and interact with a broad range of organisms that may have an impact on plant health (Mendes *et al.*, 2013; Gruden *et al.*, 2020; Pozo *et al.*, 2020). Among the many positive interactions with soil-borne organisms, the mutualistic association with arbuscular mycorrhizal fungi (AMF), known as arbuscular mycorrhizal (AM) symbiosis, deserves special attention. Due to the obligate biotrophy of these fungi, host plants are required to provide them with photosynthates and lipids for the development, maintenance and function of mycorrhizal structures (Bago *et al.*, 2000; Keymer *et al.*, 2017). In return, the AMF assist the host plant in the acquisition of water and mineral nutrients, becoming extremely important for the uptake of inorganic phosphate, nitrogen and various micronutrients (Smith and Smith, 2011; Hodge and Storer, 2015; Ezawa and Saito, 2018). In addition, AM symbiosis also result in an enhanced ability of the host plant to overcome adverse conditions, including abiotic and biotic stress factors (Sánchez-Bel *et al.*, 2016; He *et al.*, 2017; Rivero *et al.*, 2018, Fracasso *et al.*, 2020; Miozzi *et al.*, 2020; Sanmartín *et al.*, 2020a; b).

The association of plants with AMF and other beneficial microbes can stimulate the plant’s immune system, rendering the plant more resistant to the attack by different aggressors. This ‘alert’ state is known as induced systemic resistance (ISR; Choudhary *et al.*, 2007; Pieterse *et al.*, 2014). Usually, ISR implies a faster and more efficient activation of the plant defence responses upon pathogen or pest attacks. Thus, defence priming is an intrinsic part of ISR, conditioning the plants for the super activation of defences against environmental challenges (Martínez-Medina *et al.*, 2016; Mauch-Mani *et al.*, 2017). Priming is a low-cost defensive strategy since plant defences are not (or only slightly) activated in the absence of stress, but they are strongly triggered in response to a challenge (Mauch-Mani *et al.*, 2017).

The ISR achieved in mycorrhizal plants is known as mycorrhiza-induced resistance (MIR; Pozo and Azcón-Aguilar, 2007). Several reports have demonstrated the functionality of MIR in protecting plants against a wide range of belowground aggressors such as soil-borne pathogens, nematodes or root-chewing insects (Currie *et al.*, 2011; Jung *et al.*, 2012; Schouteden *et al.*, 2015; Olowe *et al.*, 2018; Pozo *et al.*, 2020). However, contrasting results have been reported regarding the effect of mycorrhiza on aboveground attackers, and the efficiency of MIR seems to be related to the lifestyle and feeding guild of the aggressors (Pozo and Azcón-Aguilar, 2007). Indeed, MIR is generally accepted to be effective mainly against necrotrophic pathogens and generalist leaf-chewing insects (Hartley and Gange, 2009; Jung *et al.*, 2012; Roger *et al.*, 2013; Song *et al.*, 2013; Nair *et al.*, 2015; Sánchez-Bel *et al.*, 2016; He *et al.*, 2017). Remarkably, those organisms are usually sensitive to the plant defences regulated by jasmonic acid (JA) signalling. Therefore, the priming of JA-dependent responses in AM plants has been proposed as the main molecular mechanism underlying MIR (Jung *et al.*, 2012; Song *et al.*, 2013; Minton *et al.*, 2016; He *et al.*, 2017).

JA signalling is a key regulator of plant defences against chewing herbivores (Erb and Reymond, 2019; Gruden *et al.*, 2020), often in synergy with abscisic acid (ABA)-related signalling (Vos *et al.*, 2013). For example, proteinase inhibitors (PI), antifeedant proteins that negatively impact the development of herbivores, as well as several toxic specialized metabolites like alkaloids or glucosinolates, are known to be induced after herbivory in a JA-dependent manner (Orozco-Cardenas *et al.*, 1993; Wasternack and Hause, 2013; Erb and Reymond, 2019). The oxylipin pathway, which is responsible of the biosynthesis of JA and its derivatives, is positively regulated by mycorrhization in tomato roots (López-Ráez *et al.*, 2010; Fernández *et al.*, 2014; Rivero *et al.*, 2015). Tomato plants colonized by the AMF *Funneliformis mosseae* negatively affected the performance of larvae from the generalist herbivore *Helicoverpa armigera* (Song *et al.*, 2013); the mycorrhizal plants displayed JA-primed accumulation and a stronger induction in the expression of several genes of the oxylipin pathway, after the herbivore attack.

Besides the study of JA and its marker genes, the potential effect of AM establishment in the rearrangement of plant secondary metabolism after herbivory, has not been explored. Most studies dealing with herbivory have focused on targeted analysis towards secondary metabolites with known toxicity against herbivores, such as some alkaloids or iridoid glycosides (Andrade *et al.*, 2013; Tomczak *et al.*, 2016). However, a global overview of the metabolic reprogramming in AM plants in response to herbivory is still missing.

Untargeted metabolomics approaches have significantly contributed to understanding the impact of AM symbiosis on the host plant. Remarkable changes on the metabolomic profile have been evidenced in mycorrhizal roots (Schliemann *et al.*, 2008; Laparre *et al.*, 2014, Rivero *et al.*, 2015; 2018, Saia *et al.*, 2015). In tomato, untargeted metabolomics revealed up-regulation of the oxylipin and phenylpropanoid biosynthetic pathways in mycorrhizal roots (Rivero *et al.*, 2015; 2018). This methodology has also been applied to identify potential primed compounds that can mediate the enhanced stress tolerance achieved in mycorrhizal plants (Rivero *et al.*, 2018, Tugizimana *et al.*, 2018). We previously identified several primed metabolites involved in the increased tolerance of mycorrhizal tomato against salinity stress, such as B6 vitamers or the flavonoid catechin (Rivero *et al.*, 2018). Similarly, phenolic compounds, lipids, and sugars have been shown to be modulated in response to drought in mycorrhizal roots (Bernardo *et al.*, 2019).

The modulation of the metabolome by AM in aboveground tissues has been less explored, and its impact seems to be highly dependent on the plant species (Schweiger *et al.*, 2014). The impact of AM on the metabolic profiles in shoots seems to be much lower than in roots. In fact, Se*necio jacobea* plants colonized by *Rhizoglomus irregulare* showed significant changes in the root metabolome while no alteration was described in the shoots (Hill *et al.*, 2018). Remarkably, a combination of untargeted and targeted metabolomics has recently allowed the identification of a group of blumenol derivates that seem to exclusively accumulate in shoots of mycorrhizal plants, independent of the plant species or any stresses (Wang *et al.*, 2018), confirming that a very limited set of compounds is consistently altered in shoots of mycorrhizal plants in the absence of stress.

Less information is available about the metabolic rearrangement in shoots of AM plants following biotic challenges. Overaccumulation of phenolic acids, amino acids, indoles and several intermediates in the oxylipin pathway were observed in leaves of tomato AM plants upon *B. cinerea* infection, suggesting that metabolic changes in the leaves also contribute to the final output of MIR (Sánchez-Bel *et al.*, 2016). Regarding interactions with herbivores, modulation of metabolic reprogramming by ectomycorrhizas has been shown in poplar, increasing its resistance to the leaf beetle *Chrysomela populi* (Kaling *et al.*, 2018). However, no information is available about the possible regulation of host plant metabolism by AM symbiosis in response to herbivory.

In the present study, we investigated whether *Funneliformis mosseae* colonization in tomato plants has a negative impact on the common pest *Spodoptera exigua*. To explore the potential molecular mechanisms regulating such effects, it should be considered that the plant defences triggered differ according to the proximity to the damaged area (Koo *et al.*, 2009). In fact, the defensive response (such as phytohormone signalling activation or accumulation of toxic metabolites) differs between damaged tissues (local response) and those still non-attacked (systemic response) that may prepare the plant for a potential subsequent aggression (Dugé de Bernonville *et al.*, 2017). Thus, an in-depth study of the metabolic response to herbivory requires a separate analysis of the local and systemic responses. In this study we compared the local and systemic responses to herbivory by *S. exigua* in mycorrhizal and non-mycorrhizal plants following an untargeted metabolomic approach. We identified primed accumulation of defensive compounds locally in the damaged leaf tissues, which may be responsible for the enhanced ability of AM plants to cope with this aggressor.

## Materials and methods

### Plant and fungal materials and growing conditions

The AMF *Funneliformis mosseae BEG12* (*F. mosseae*, formerly *Glomus mosseae*, International Bank of Glomeromycota, https://www.i-beg.eu/cultures/BEG12.htm), was maintained in an open-pot culture of *Trifolium repens* L. mixed with *Sorghum vulgare* Pers. plants in a greenhouse. The inoculum consisted of substrate (vermiculite/sepiolite, 1:1), spores, mycelia and infected root fragments from those cultures. Tomato seeds (*Solanum lycopersicum* L. ‘Moneymaker’) were surface sterilized by immersion in 4% NaHClO (10 min) containing 0.02% (v/v) Tween20 ®, rinsed thoroughly with sterile water and incubated for seven days in an open container with sterile vermiculite at 25 ºC. Tomato plantlets were transferred to 250 ml pots containing a sterile sand: vermiculite (1:1) mixture. Pots for mycorrhizal treatments were inoculated by adding 10% (v/v) *F. mosseae* inoculum. Uninoculated plants received an aliquot of a filtrate (<20 µm) from *F. mosseae* inoculum in order to provide the general microbial population, but free of AMF propagules.

A total of ten plants were used for each treatment, randomly distributed and grown in a greenhouse at 24 °C/16 ºC with a 16 h/8 h diurnal photoperiod and 70% humidity. Plants were watered three times a week with Long Ashton nutrient solution (Hewitt, 1966) containing 25% of standard phosphorus (P). Plants were harvested after eight weeks, the fresh weight of shoots and roots was determined, and the leaf material immediately frozen in liquid nitrogen and stored at −80 ºC. An aliquot of each individual root system was reserved for mycorrhizal quantification.

### Determination of mycorrhizal colonization

Mycorrhizal colonization was estimated after clearing washed roots in KOH (10%) and subsequent staining of fungal structures with 5% ink (Lamy, Germany) in 2% acetic acid (Vierheilig *et al.*, 2005). The extent of mycorrhizal colonization (expressed as percentage of total root length colonized by the AMF) was calculated according to the gridline intersection method (Giovannetti and Mosse, 1980) using an Eclipse 50i microscope (Nikon, Japan) and brightfield conditions.

### Insect rearing and herbivore performance set-up in tomato plants

**Fig. 1.**
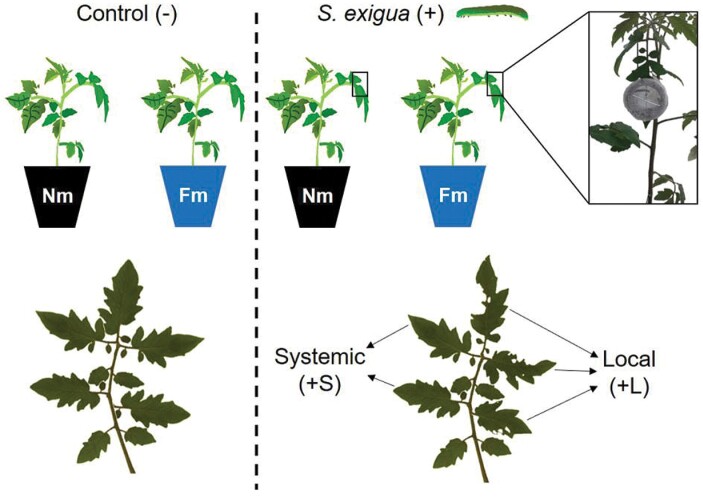
Experimental set-up and samples used. Harvested shoot material were from *Funneliformis mosseae*-colonized (Fm) or non-mycorrhizal (Nm) tomato plants, infested (+) or not (-) with *Spodoptera exigua*. Two second instar larvae were placed into a clip-cage per plant. Clip-cages were moved every day into new leaflets and larval mortality was recorded daily, for two weeks. At the end of the assay, shoot material was collected from each treatment, differentiated into leaflets from non-stressed plants (control; -), leaflets with damage from *S. exigua* feeding (local, +L) and those leaflets without damage but contiguous to where *S. exigua* fed (systemic, +S).

Batches from the same *S. exigua* (Lepidoptera: Noctuidae) laboratory colony were used for the different experiments. The colony was established with eggs provided by Andermatt Biocontrol AG (Grossdietwil, Switzerland) and reared in the laboratory for more than 50 generations. Larvae were reared on artificial diet (Greene *et al.*, 1976) at 25±3 ºC with 70±5% relative humidity and a 16 h/8 h diurnal photoperiod. For the *in planta* experiments, once they reached second instar stage of development, two larvae were placed on a leaflet from the third true leaf of each six week-old tomato plant, using 30 mm Ø clip-cages to limit the feeding area and avoid their escape. Clip-cages were moved into new leaflets every two days to make sure they always had food available. Infestation was maintained for two weeks. To monitor insect development, larvae biomass and mortality were measured every day. Dead larvae were replaced by new ones in order to keep the same stimulus in the plant. Finally, eight week-old plants with two weeks of herbivory infestation were harvested. Leaflets from non-infested plants (control, –), leaflets from infested plants where *S. exigua* directly fed (local, +L) and leaflets contiguous to those with direct damage (systemic, +S) were harvested separately and immediately frozen in liquid nitrogen to be stored at –80 °C until use for molecular analysis (Fig. 1).

### Liquid chromatography and electro-spray ionization (LC-ESI) mass spectrometry

#### LC–ESI full scan mass spectrometry

Freeze-dried leaves (50 mg) were homogenized on ice in 1 ml of MeOH:H_2_O (10:90) containing 0.01% of HCOOH. The homogenate was centrifuged at 15 000 × *g* for 15 min at 4 ºC, and the supernatant was recovered and filtered through 0.2 μm cellulose filters (regenerated cellulose filter, 0.20 μm, 13 mmØ. pk/100; Teknokroma, St Cugat, Spain). Subsequently, 20 μl of the filtered supernatant was injected into an Acquity ultra-performance liquid chromatography system (UPLC; Waters, Mildford, MA, USA), which was interfaced with a hybrid quadrupole time-of-flight equipment (Q-TOF-MS Premier, Waters, Mildford, MA, USA). Analytes were eluted with an aqueous methanol gradient containing 0.01% HCOOH. Solvent gradients and further chromatographic conditions were performed as previously described (Agut *et al.*, 2014). Five biological replicates were randomly injected for every treatment. LC separation was performed using an ultra-performance liquid chromatography (UPLC) Kinetex C18 analytical column with a 5 μm particle size, 2.1 × 100 mm (Phenomenex, Madrid, Spain). To accurately identify the signals detected, a second fragmentation function was introduced into the TOF analyser. This function was programmed in a t-wave ranging from 5 to 45 eV to obtain a fragmentation spectrum of each analyte (Agut *et al.*, 2014; Gamir *et al.*, 2014).

To precisely identify metabolites, a library of plant metabolites was generated using chemical standards. The compounds from this library were characterized at the level of retention time, exact mass, and spectrum fragmentation (Schymanski *et al.*, 2014). Up to 84 compounds were prepared at a final concentration of 100 ppb in a composite solution (Rivero *et al.*, 2015) and injected through the UPLC in both positive and negative electro-spray ionization (ESI^+^; ESI^−^) modes. For those compounds that were not represented in the internal library, the signals obtained in the untargeted metabolomic analysis were confirmed by comparing the fragmentation spectrum in the Massbank, Metlin or Human Metabolome databases (www.massbank.jp; www.masspec.scripps.edu; www.hmdb.ca).

#### Full scan data analysis

Data were acquired in centroid mode and subsequently transformed into .cdf files using the Databridge from MassLynx 4.1 software (MassLynx 4.1, Waters, USA). Chromatographic signals were processed using the software *R* for statistical purposes. Signals from positive and negative electrospray ionization (ESI^+^; ESI^-^) were processed separately. Peak peaking, grouping and signal corrections were performed using the XCMS algorithm (Smith *et al.*, 2006). A total of 1132 signals were obtained, 793 in ESI^+^ mode and 339 ESI^-^ mode (Supplementary Table S1). Metabolite amounts were analysed based on normalized peak area units relative to the dry weight. Adduct, isotope correction, and Kruskal–Wallis test (*P*<0.05) were performed by using the MarVis Suit 2.0 software tool (Kaever *et al.*, 2015). To determine a global behaviour of the signals, data obtained from positive and negative ESI were combined using MarVis Suit 2.0 software. Subsequently, data were normalized by sum, transformed by cube root, and scaled by Pareto method in order to obtain the sparse partial least squares discriminant analysis (sPLSDA) and heatmap plots, which were generated using MetaboAnalyst 4.0 software (www.metaboanalyst.ca), a comprehensive web-based package for a range of metabolomics applications (Chong *et al.*, 2019).

### Antiherbivore properties of selected compounds

Metabolites with primed accumulation in mycorrhizal plants upon herbivory were selected from the metabolome analyses, and those commercially available were purchased to test their effect on *S. exigua* larvae mortality in different experimental set ups. Treatments were: (i) physostigmine (PHYS; AnalytiCon Discovery GmbH, Potsdam, Germany); (ii) azelaic acid (AZA; Sigma-Aldrich, Germany); (iii) 4-oxododecanedioic acid (4-OXO; Biocrick, Chengdu, China); and (iv) feruloylputrescine (FP; AKos Consulting & Solutions Deutschland GmbH, Steinen, Germany).

#### *In planta* bioassay

Non-mycorrhizal tomato plants (*Solanum lycopersicum* L. ‘Moneymaker’) were grown in 500 ml pots containing a sterile sand: vermiculite (1:1) mixture under greenhouse conditions, as described above. Chemical treatments were applied five weeks after transplanting by spraying the third true leaf of each plant (*n=*12) with an aqueous solution containing 100 ppm of the compounds, 2% MeOH and 0.02% Tween 20 ®. Control plants were mock-treated with the same solution. For each plant and treatment, two *S. exigua* larvae at the third instar stage (previously reared on artificial diet) were placed on two different leaflets from the treated leaf using 30 mm Ø clip-cages. Larvae that did not feed in the first 24 h were discarded. Larval mortality was monitored every 72 h during the following six days.

#### Bioassay on artificial diet

*S. exigua* larvae were reared on artificial diet (Hoffman and Lawson, 1964) until they became second instar. Sixteen newly molted second instar larvae were transferred to diet supplemented with the different selected compounds at a final concentration of 30 ppm or 3 ppm (from stock solutions in methanol or water). Control larvae were transferred to diet containing the same concentration of the solvent used for the preparation of the supplemented diets (0.6% methanol). For those compounds dissolved in water (AZA and FP) methanol was added to the diet to a final concentration of 0.6% to get an equivalent concentration of methanol in all the treatments. In addition, a group of larvae were also treated with a mixture of the four tested compounds (final concentration of 3 ppm for each compound). Larval mortality was recorded daily during the following 6 d. Sixteen larvae were used per dose and treatment, and the bioassay was repeated twice.

### Statistical analyses

Besides the methods and software for metabolomic analysis described above, all statistical analyses (two-way ANOVAs and post hoc tests applied when appropriate, as indicated in the corresponding figure legends) were conducted using Statgraphics Plus 3.1 (Rockville, MD, USA), *R* software version 2.9.2 (R Development Core Team) and the XCMS package. Larval survival distribution comparisons between treatments were performed using the Logrank (Mantel-Cox) non-parametric test. The reported error bars represent the SE of the survival fractions. Statistical analysis and graphs were generated with GraphPad Prism v.7.0.1.

## Results

### Arbuscular mycorrhizal symbiosis increases mortality of *S. exigua* larvae

**Fig. 2.**
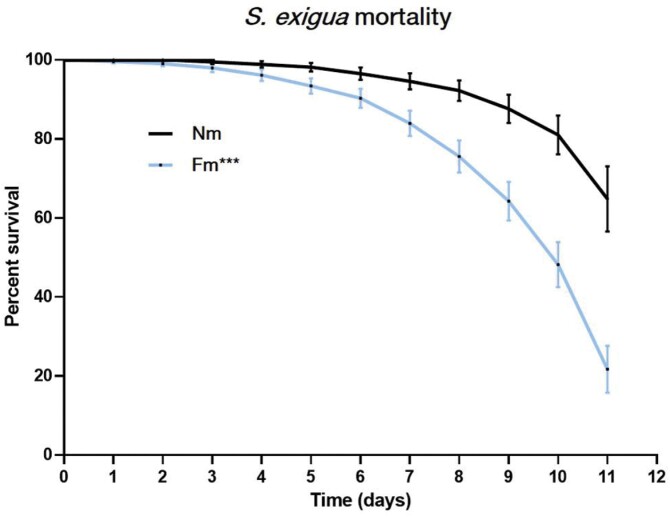
Percentage survival of *Spodoptera exigua* larvae fed on non-mycorrhizal plants (Nm, black line) or on plants colonized by AMF *Funneliformis mosseae* (Fm, blue line). Tomato plants colonized or not colonized by *F. mosseae* (*n*=20) were infested with second instar *S. exigua* larvae (two per plant) using clip cages, and mortality was recorded daily. Asterisks indicate statistically significant differences between treatments [***P <0.0005, Logrank (Mantel-Cox)].

In order to determine whether root colonization by *F. mosseae* affects *S. exigua* performance, tomato plants displaying a well-established mycorrhizal symbiosis were challenged with *S. exigua* larvae. The percentage of root colonization by the mycorrhizal fungus was ~13% by the end of the experiment (Supplementary Fig. S1A). The symbiosis had no effect on plant growth (Supplementary Fig. S1B). This is of interest for studies on defence mechanisms, by avoiding the potential effects of improved plant growth on insect performance. AM establishment was clearly detrimental to the development of *S. exigua*. Larvae fed on *F. mosseae*-colonized tomato plants (Fm) showed a higher mortality than those feeding in non-mycorrhizal plants (Nm; Fig. 2). In fact, 50% of the larvae in Fm plants were dead by day 10, while the mortality was only 15% for larvae on Nm plants.

Despite the differences in the gain of weight of the survivors between Fm and Nm plants not being statistically significant (*P*>0.05), there was a clear trend showing reduced larval weight gain in Fm plants (Supplementary Fig. S1C). In addition, 11 days following infestation, 17% of the larvae fed on Nm plants started to pupate, while in Fm plants none of the larvae reached this developmental stage.

### *S. exigua* herbivory had an important impact on the leaf metabolome

**Fig. 3.**
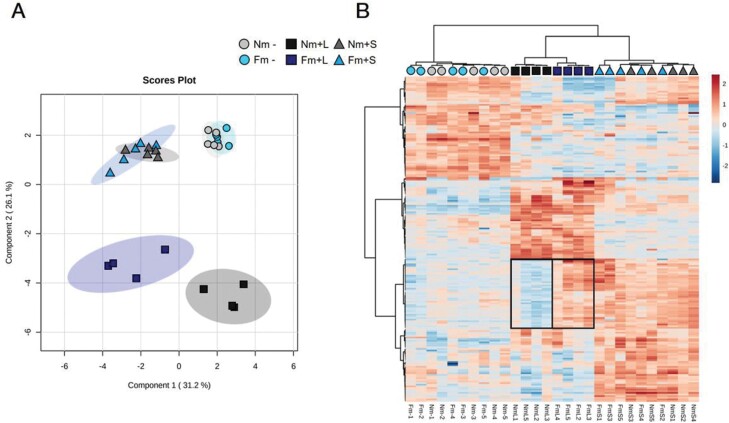
Overview of the metabolomic reprogramming in tomato leaves subjected to *Spodoptera exigua* feeding for 15 days. Six weeks old tomato plants non-mycorrhizal (Nm) or colonized by the arbuscular mycorrhizal fungi *Funneliformis mosseae* (Fm) were infested with S*. exigua* second instar larvae, and leaves were harvested after two weeks of systemic herbivory. Lyophilized leaf material from control non-infested plants (-), infested leaflets (local response to herbivory, +L) and non-damaged leaflets from infested plants (systemic response to herbivory, +S) were analysed through ultra-high performance liquid chromatography interfaced with a quadrupole time-of-flight mass spectrometer (UPLC-Q-TOF-MS), in order to monitor metabolomic changes. Signal intensity was determined in all samples after normalizing the chromatographic area for each compound to the dry weight of the sample. All 1132 registered signals (Supplementary Table S1), from combination of both positive and negative electrospray ionization (ESI), were compared using non-parametric Kruskal–Wallis test, and only data with significant differences between groups at *P*<0.05 were used for the supervised analysis (n=5 for - and +S, n=4 for +L), obtaining 200 signals (Supplementary Table S2). Grey and blue colour scales represent Nm and Fm treatments, respectively, and for each case, circles represent non-stress (-), dark coloured squares represent changes in infested, damaged tissue (L), and triangles represent changes in systemic responses to herbivory (S). (A) Supervised three-dimensional sparse partial least squares discriminant analysis (sPLSDA) representation of the major sources of variability. (B) Heatmap and clustering representation of the 200 signals showing differences among treatments. Cluster of metabolites showing an over-accumulation pattern upon herbivory in L tissue only in Fm plants (priming pattern) is highlighted.

We hypothesized that the reduced larval survival rate and performance in Fm plants compared with non-colonized ones was a result of changes in the defence response in the mycorrhizal plants. To test this possibility, we compared the plant responses of Nm- and Fm-colonized plants subjected to (+) or not subjected to (–) *S. exigua* feeding during 14 days, through an untargeted metabolomic analysis. To have a more precise overview of the metabolic regulation upon herbivory, we harvested the leaflets where the larvae fed separately from the other unwounded leaflets of the same leaves. Thus, herbivory-related changes were explored in local wounded leaflets (+L) or in unwounded leaflets (systemic response, +S), as shown in Fig. 1. A total of 1132 signals (potential compounds) were registered (Supplementary Table S1), and subsequent statistical analysis showed 200 signals with significantly different accumulation in at least one of the different treatments (*P*<0.05, Supplementary Table S2). A representation of a supervised analysis (sPLSDA) of these 200 signals revealed that, as expected, herbivory had a strong impact on plant metabolism (Fig. 3A). According to the two main components contributing to data variation, the differences were more pronounced locally at the feeding sites than systemically in leaflets that were not exposed to direct herbivory, since the group of these signals were more distant compared with undamaged leaves (Fig. 3A). These observations were further confirmed by a heatmap analysis (Fig. 3B). This analysis clearly showed the rearrangement of the leaf metabolomic profile following herbivory. Remarkably, in the absence of herbivory (Nm– versus Fm–), AM symbiosis had very low impact on the foliar metabolic profile, since only one hit out of the 1132 detected displayed significantly altered accumulation (*P*<0.05) in mycorrhizal plants (Supplementary Fig. S2A, B). Although still not fully characterized, exact mass identification of this metabolite matched with 11-carboxyblumenol C-9-O-Glc, a metabolite recently reported as a shoot marker of AM symbiosis (Wang *et al.*, 2018).

### *Funneliformis mosseae* colonization primes the accumulation of specific metabolites in leaves damaged by *S. exigua*

To explore potential changes in the chemistry of the leaves leading to enhanced larval mortality in mycorrhizal plants, we compared the metabolic profile of mycorrhizal and non-mycorrhizal plants upon herbivory. Few changes in the metabolite profiles were observed between Nm and Fm in the systemic response to herbivory (Nm+S versus Fm+S), as confirmed by the overlap in both sPLSDA and heatmap plots (Fig. 3A, B). In contrast, the local responses to herbivory were modulated as illustrated by the clear separation between the Nm+L and Fm+L samples in the sPLSDA (Fig. 3A). The heatmap analysis confirmed these clearly separated profiles, while uninfested Nm and Fm, and systemic Nm+S and Fm+S clustered together. The distinct pattern of metabolite accumulation in Fm+L revealed a set of compounds with a stronger accumulation, resembling a priming profile (highlighted by a black square, Fig. 3B). In contrast, these metabolites were not modulated by the presence of AM symbiosis in the absence of herbivory. Intriguingly, as shown by their similar patterns in the heatmap, most of those compounds seemed to accumulate to similar levels in systemic tissues of both Nm and Fm plants (Nm+S, Fm+S).

### Characterization of the metabolic pathways differentially regulated in response to herbivory

**Fig. 4.**
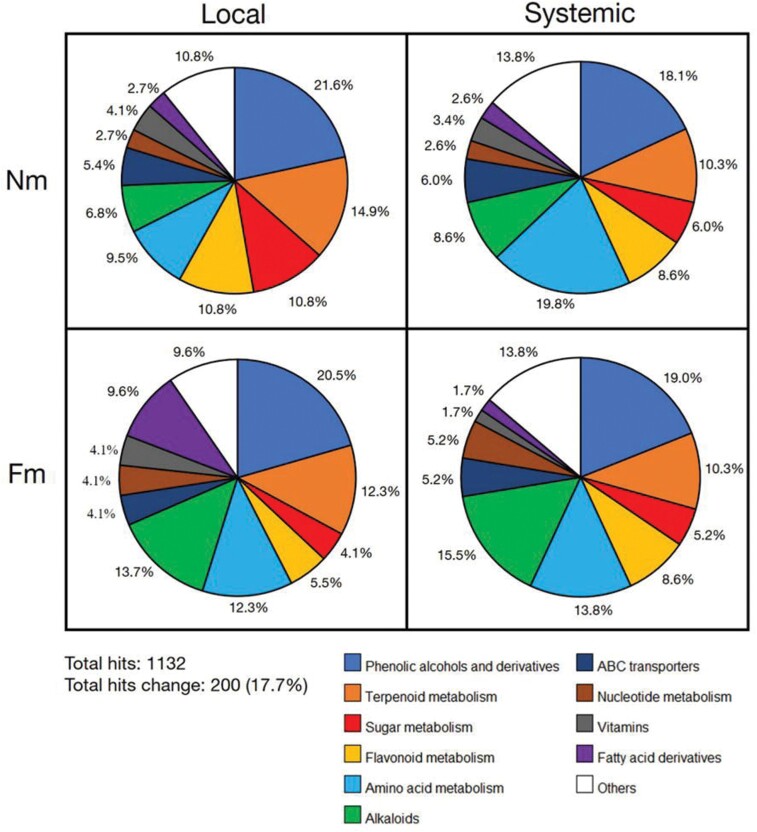
Composition of altered metabolic pathways from tomato plants leaves under *S. exigua* herbivory. Six weeks old tomato plants non-mycorrhizal (Nm) or colonized by the arbuscular mycorrhizal fungi *Funneliformis mosseae* (Fm) were infested with S*. exigua* second instar larvae, and leaves were harvested after two weeks of systemic herbivory. After untargeted metabolomic analysis through ultra-high UPLC-Q-TOF-MS (described in Fig. 3 legend), obtained signals were compared using non-parametric Kruskal Wallis test (*P*<0.05), and only data with differences between herbivory treatments (local or systemic) and non-stress conditions were used for the supervised analysis (n=5 for - and +S, n=4 for +L). The resulting hits were identified by exact m/z and/or spectra coincidence and subsequently grouped into the different *S. lycopersicum* metabolic pathways from KEGG databases included in software MarVis 2.0. Finally, the pie charts with the percentage of main metabolic pathways altered in each treatment were represented.

Following untargeted metabolomic analysis, we aimed to identify the 200 signals (Supplementary Table S2) whose accumulation differed in response to herbivory by exact m/z and/or individual spectrum match with databases. For such classification, we used the *S. lycopersicum* KEGG and internal databases, putatively identifying 107 signals by m/z or/and fragmentation spectrum (Supplementary Table S3). Subsequently, we classified them into the major metabolic pathways (Supplementary Fig. S3), representing the percentage of differentially regulated metabolites in each of these categories for each treatment condition (Fig. 4**).** This classification illustrates that the local response to *S. exigua* herbivory resulted in altered accumulation of compounds in multiple pathways, but this consisted mostly of phenolic compounds (21%), terpenoids (14.9%), carbohydrates (10.8%), and flavonoids (10.8%). In distal leaflets, the systemic response resulted in activation of those pathways too, but with major changes in metabolites related to amino acid metabolism. In mycorrhizal plants, these response patterns were different. In local damaged leaflets Fm plants showed a higher proportion of metabolites within the fatty acid metabolism (9.6%) and alkaloids (13.7%) than Nm plants, where these categories only reached 2.7% and 6.8%, respectively (Fm+L versus Nm+L). In contrast, the proportion of herbivore-modulated compounds related to sugar and flavonoid metabolism was lower in Fm+L than in Nm+L samples. Regarding the systemic responses, the proportion of alkaloids and metabolites related to nucleotide metabolism was almost doubled in Fm (Fm+S) compared with Nm plants (Mm+S).

Thus, these results illustrate that mycorrhization implies mostly a stronger activation of compounds related to alkaloid and fatty acid metabolism upon herbivory.

### Mycorrhiza-primed metabolites in local response to *S. exigua* herbivory

**Fig. 5.**
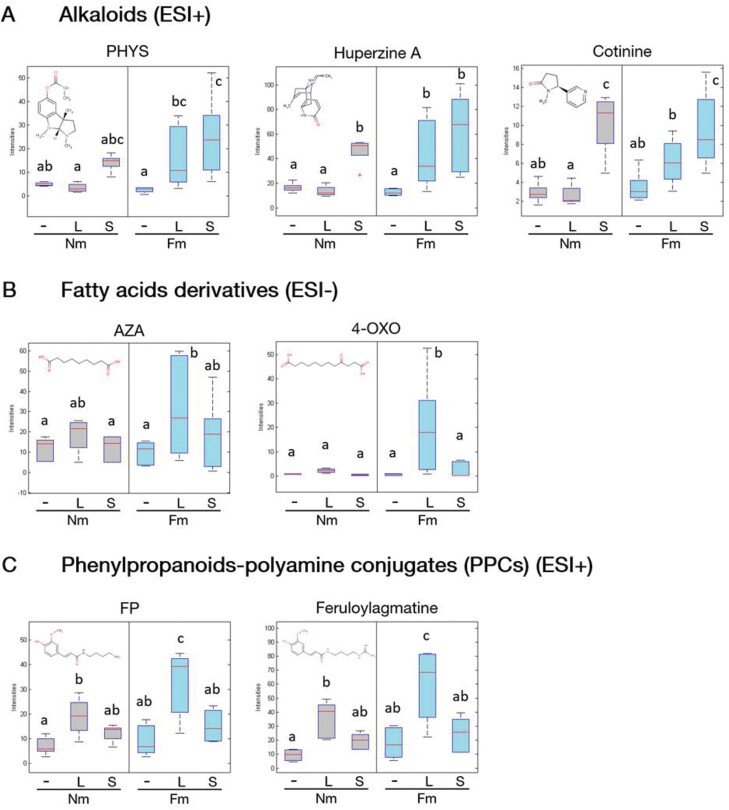
Boxplots of selected metabolites with a primed accumulation pattern in response to local herbivory. Six week-old tomato plants, previously colonized by arbuscular mycorrhizal fungi (AMF) *Funneliformis mosseae* (Fm) or not (non-mycorrhizal, Nm), were infested with second instar larvae of S*. exigua*, and infestation maintained for two weeks more. Leaves from plants in the absence of stress, or with local (L) or systemic (S) damage by *S. exigua* feeding were analysed through an untargeted metabolic assay in order to monitor metabolic changes (data acquisition as described in Fig. 3 legend). Grey colour represents accumulation in Nm plants and blue colour represents accumulation in Fm-colonized plants. Boxes represent the interquartile range, thick red-lines represent the median, whiskers represent maxima and minima within 1.5 times the interquartile range, and red-crosses show outliers. Putative metabolites were classified into three main metabolic groups: (A) alkaloids family; (B) fatty acids derivatives family; and (C) phenylpropanoid-polyamine conjugates (PPCs) family. Two-way factorial ANOVA (using Fm colonization and herbivory treatments as factors) were performed. Data not sharing a letter in common differ significantly according to the Fisher’s LSD test (*P*<0.05, *n*=6).

As described above, the most remarkable difference between the response to herbivory in Nm and Fm plants is the cluster of compounds displaying a primed accumulation pattern in the damaged tissues (Fig. 3B, highlighted cluster). Identification through exact mass and/or fragmentation revealed alkaloids, fatty acid derivatives and phenylpropanoid-polyamine conjugates (PPCs) among the metabolites within this primed cluster (Table S3, Fig. 5A-C). The alkaloids, including physostigmine (PHYS, also known as eserine), huperzine A and cotinine, appeared to be over accumulated locally after larvae feeding only in Fm plants, while remaining unaltered locally in Nm plants. Interestingly, the priming profile of these compounds was only observed in local tissues, since they accumulated in systemic leaves upon herbivory in both Nm and Fm plants (Fig. 5A).

Among the fatty acid derivatives showing a primed accumulation profile in Fm leaves, we found azelaic acid (AZA) and 4-oxododecanedioic acid (4-OXO). These compounds, unlike alkaloids, were not altered at all in non-mycorrhizal plants, and only marginally in systemic leaves of mycorrhizal plants (Fig. 5B).

Finally, a group of phenylpropanoid-polyamine conjugates (PPCs), also known as hydroxycinnamic amides or phenolamides, formed by condensation of phenolic acids with amines such as polyamines and arylamines, were found to be primed in local leaves of Fm plants (Fig. 5C). This was the case for feruloylputrescine (FP) and feruloylagmatine that showed significantly higher (*P*<0.05) accumulation only in Fm leaves in response to herbivory (Fm+L). In contrast, other compounds in this group, tricoumaroylspermidine and feruloyl-2-hydroxyputrescine, besides being locally accumulated in response to herbivory in Fm plants, were also accumulated in systemic tissues, regardless of the mycorrhizal status of the plant (Supplementary Fig. S4).

### Effect of selected primed compounds on *S. exigua* mortality

**Fig. 6.**
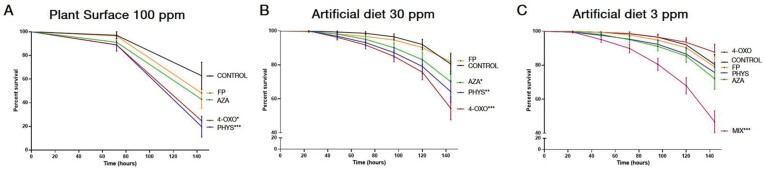
Antiherbivore effect of selected compounds. (A) Survival of *Spodoptera exigua* larvae when fed on plants sprayed with 100 ppm each of physostigmine (PHYS), azelaic acid (AZA), 4-oxododecanedioic acid (4-OXO), feruloylputrescine (FP), or a mixture (MIX) of the four compounds. (B) Survival of *Spodoptera exigua* larvae when fed on artificial diet supplemented with 30 ppm each the different compounds listed in (A). (C) Survival of *Spodoptera exigua* larvae when fed on artificial diet supplemented with 3 ppm each of the different compounds listed in (A). For each curve, 12 plants, with two larvae each were used with the different compounds (*n*=16). Larval mortality was recorded every 72 h (A) or daily (B, C) during the following 6 d. For (B) and (C), bioassays were repeated twice. Asterisks indicate statistically significant differences between control and compound-fed samples (Logrank (Mantel-Cox). *P<0.05, **P<0.005, ***P <0.0005, ****P<0.00005.

Four of the main primed compounds were tested for their effects on insect survival by applying the synthetic compounds (PHYS, AZA, 4-OXO, and FP) to the plant surface as well as in combination with an artificial diet. Both experimental set ups revealed a significant detrimental effect (*P*<0.05) on larvae for the PHYS and the 4-OXO treatments (Fig. 6A, B). In addition, AZA also led to a reduction in larvae survival, but only when combined with artificial diet. No effect of FP was observed for any of the treatments. These results confirm the antiherbivore properties of most of the primed compounds tested, and suggest that their action occurs by direct negative effects on the herbivore and not through the activation of antiherbivore defences in the plant, as the addition of the compound in artificial diet, in the absence of plant tissues, was sufficient for increasing larval mortality.

The assay on artificial diet was carried out at two different doses of the primed compounds (30 ppm and 3 ppm). At the lower dose, none of the compounds applied individually had an impact on larval survival (Fig. 6C). However, the simultaneous addition of the four compounds into the diet resulted in a significant increase (*P*<0.0005) in larval mortality compared with the non-supplemented diet, suggesting a potential synergistic effect of the active compounds.

## Discussion

In the present study, we demonstrated that tomato root colonization by the AMF *F. mosseae* has a significant impact on the host interaction with the generalist pest *S. exigua*. The larvae feeding in these mycorrhizal plants showed an increased mortality and delayed development (Fig. 2), thus these plants are more efficient in containing the herbivore. Although the protective role of AM against pathogens and the underlying molecular mechanisms have been studied previously (Nair *et al.*, 2015; Sánchez-Bel *et al.*, 2016; Jung *et al.*, 2012; Sanmartín *et al.*, 2020a; b), the influence of symbiosis against insects at the mechanistic level are less studied (Song *et al.*, 2013; Minton *et al.*, 2016). Contrasting effects have been reported against leaf chewers, explained as the result of the potential positive effects of symbiosis on the larvae by increasing the plant nutritional value, or the negative effects in the case of improved plant defences (Pozo *et al.*, 2020). Indeed, it has been proposed that AM plants display an optimal tuning of their defences, leading to improved resistance.

Mycorrhization has been reported to decrease the performance of the generalist chewing insects *Helicoverpa armigera* (Song *et al.*, 2013) and several *Spodoptera* spp. (Roger *et al.*, 2013; He *et al.*, 2017). Mycorrhizal protection against herbivores (*Spodoptera litura*) was shown even to be transferred to neighbouring plants by warning them through common mycorrhizal networks (Song *et al.*, 2014). However, benefits in larval fitness from the generalist *Mamestra brassicae* were observed on mycorrhizal *Plantago*, despite the strongest induction of aucubin (toxic metabolite against herbivores) in AM-plants (Tomczak *et al.*, 2016). The authors hypothesized that this effect could be due to the better plant quality found in mycorrhizal plants. Mycorrhizal rice also exhibited higher susceptibility to the generalist *Spodoptera frugiperda*, where mycorrhization resulted in plant-growth promotion (Bernaola *et al.*, 2018). In our experimental system there were no differences in plant growth in response to the mycorrhizal symbiosis, so that we could evaluate, without the interference of significant plant growth effects, the contribution of the plant defence responses to the impact of mycorrhiza on the larvae.

A strongest defensive capacity in AM plants has been shown in previous studies, usually through phytohormone measurements or quantification of metabolites with known toxic effects against insects (Andrade *et al.*, 2013; Song *et al.*, 2013; Minton *et al.*, 2016; Tomczak *et al.*, 2016; Sanmartín *et al.*, 2020b). However, a more holistic overview of the possible modulation of metabolism in the host plant is missing. Here, by using untargeted metabolomics analysis we attempted to profile the metabolic tuning in Nm and Fm plants when interacting with *S. exigua*, aiming to uncover the mechanisms underlying the enhanced mortality of the larvae in Fm plants.

In previous studies, we showed that AMF colonization resulted in a strong metabolic rearrangement in tomato plant roots in absence of stress (Rivero *et al.*, 2015; 2018, Sanmartín *et al.*, 2020b). Here, we found a minimal alteration of the metabolome in aboveground tissues. Indeed, only one out of 1132 signals registered during the analysis showed differential accumulation in AM plants. The levels of the compound were not altered by herbivory. Its tentative exact mass identification matches with 11-carboxyblumenol C-9-O-Glc, recently reported as a mycorrhization marker in shoots (Supplementary Fig. S2; Wang *et al.*, 2018).

When examining the effect of *S. exigua* feeding on the metabolome, the bidimensional sPLSDA analysis showed that plant responses upon herbivory clearly resulted in a rearrangement of the metabolic profiles in local and systemic leaves, compared with those from uninfested plants (Fig. 3A). These modifications were different, depending on the distance to the damaged area (local or systemic responses). These distinct responses are in agreement with previous reports, where metabolic differences between damaged and non-attacked tissues were also found (Dugé de Bernonville *et al.*, 2017).

When comparing the profiles in response to herbivory in Fm and Nm plants, differences were only observed in damaged leaflets, while the systemic responses were similar in Nm and Fm plants. The heatmap exhibited a cluster of compounds with a clear primed accumulation profile in the local response to herbivory (Fig. 3B), including several metabolites accumulated to a higher level in Fm plants than in non-colonized ones. Strikingly, this cluster showing a primed profile locally upon herbivory in Fm plants resembled the systemic response. The stronger accumulation of compounds in the damaged area, together with the absence of significant modulation under non-stress conditions, reinforced the hypothesis of defence priming as being responsible for the enhanced mortality observed: the modulation of plant metabolism only takes place in response to herbivory challenge.

A deeper characterization of the response to herbivory revealed phenylpropanoids, terpenoids, flavonoids, sugars and amino acid metabolic pathways as the major groups altered in the local responses to damage by *S. exigua* attack in both Nm and Fm plants (Fig. 4). In systemic tissues, the same metabolic pathways were targeted, but the proportion of the amino acids and alkaloids was higher. The increase in alkaloids as a local and systemic response to damage was more pronounced in mycorrhizal plants. Considering the insecticidal properties of these compounds, it is tempting to speculate their potential role in the observed protection. In addition, there was a marked 3-fold higher accumulation of fatty acid-related compounds in the local response of Fm plants. This group could be also of great importance for plant defences since it comprises JA precursors from oxylipin biosynthetic pathway, among others.

We focused on those compounds showing a priming profile in mycorrhizal plants, as they could be related to the higher mortality of the larvae feeding on these plants. The three over-represented families of compounds within this cluster were alkaloids (Fig 5A), fatty acid derivatives (Fig 5B), and PPCs (Fig. 5C). Alkaloids are a well characterized group of compounds with toxic effects against herbivores (Mithöfer and Maffei, 2016). Besides their strong clear accumulation induction in local damaged leaves of Fm plants, alkaloids also showed high accumulation in systemic leaves, suggesting a higher mobility of these compounds that may contribute to improved defensive responses against subsequent infestations in distal tissues. Amongst them, cotinine, a common metabolite from nicotine catabolism (Saremba *et al.*, 2018), was found. In addition, two acetylcholinesterase inhibitors were found: PHYS and huperzine A (Luo *et al.*, 2010; Rattan, 2010).

Different studies have addressed the involvement of fatty acids as chemical signals triggering defences during negative interactions (Lim *et al.*, 2017). In our experiment we found a higher local accumulation of AZA in Fm plants upon herbivory, and 4-OXO was exclusively found as a local response to herbivory in those plants. AZA was previously reported to accumulate upon *Spodoptera littoralis* herbivory in maize (Marti *et al.*, 2013. While no antiherbivore properties have been described for AZA, it has been proposed to be a component of plant systemic immunity regulating defence priming (Jung *et al.*, 2009). Interestingly, exogenous application of AZA in tobacco cells resulted in primed expression of genes involved in systemic acquired resistance, and enhanced synthesis of PPCs (Djami-Tchatchou *et al.*, 2017), supporting the role of AZA in the priming of this family of secondary metabolites. As far as we know, no direct effect of AZA nor 4-OXO acid on plant pathogens or pests has been reported.

Lastly, PPCs (also known as phenolamides) are a diverse class of secondary metabolites present ubiquitously in plants. In our conditions, both tricoumaroylspermidine and feruloyl-2-hydroxyputrescine metabolites showed a similar pattern than alkaloids, with primed-behaviour in Fm plants locally, but high accumulation in the systemic tissues in both Nm and Fm plants. Conversely, feruloylagmatine and FP only over accumulated locally in Fm plants, with their levels remaining unaltered in distal tissues. These compounds were suggested to play an important role in plant development and defence (Edreva *et al.*, 2007; Macoy *et al.*, 2015), especially after challenge with pathogens (López-Gresa *et al.*, 2011; Yogendra *et al.*, 2015) or virus (López-Gresa *et al.*, 2016). Regarding the role of PPCs in anti-herbivore defence, an enhanced growth of *S. littoralis* reared on *Nicotiana* mutant plants lacking PPCs was reported (Kaur *et al.*, 2010). In the same study, *Nicotiana* plants sprayed with caffeoylputrescine reduced *Manduca sexta* larval growth, suggesting that PPCs are important players in plant defence against leaf chewers.

To test the potential role of the identified primed compounds in the defence response, we selected commercially available compounds representative of these groups and performed different bioassays (Fig. **6**). Plant treatments with the alkaloid physostigmine or the fatty acid derivative 4-OXO acid significantly enhanced larval mortality. To test whether this was due to a direct effect on the larvae, rather than to a defence elicitation role, we tested the effect of the compounds on artificial diet (Fig 6B,C). Both PHYS and 4-OXO acid also had a negative effect when provided an artificial diet. In addition, AZA showed a negative effect on the larvae too, but only in artificial diet. Accordingly, the data confirm a direct negative effect of the compounds on larval performance, although we cannot rule out some eliciting activity of plant defences. No effect on mortality was found for FP treatments in any of the bioassays. However, we cannot rule out indirect effects through interaction with other toxic compounds, or by altering larvae immunity as it has been reported in other systems (Bandoly *et al.*, 2016). It is remarkable that treatments with the individual compounds at lower doses (3 ppm) did not have any effect on larval survival, but their combination had a significant deleterious effect, pointing to potential synergistic effects that would support a stronger effect *in planta*, as several defensive metabolites co-occur. In fact, recent meta-analyses pinpoint to the relevance of synergistic effects of phytochemical mixtures on generalist herbivores (Richards *et al.*, 2016).

In conclusion, our results indicate that *F. mosseae* colonization of tomato roots increase plant resistance against *S. exigua.* Our study shows evidence that AM symbiosis modulates host metabolism in response to herbivory, leading to a primed accumulation of defensive compounds including fatty acid derivatives, alkaloids and PPCs. The functional analysis of some of these compounds as PHYS and 4-OXO demonstrate their negative effect on larval survival. Thus, the higher accumulation upon herbivory of these defensive compounds in mycorrhizal plants likely contributes to the enhanced larval mortality. Future studies will aim to uncover the transcriptional and post-transcriptional regulation and key regulatory elements underlying this primed accumulation of defences. Our study contributes to the identification of new functional compounds with anti-herbivore properties and illustrates that mycorrhizal colonization can prime plant metabolic responses to herbivore attack.

## Supplementary data

The following supplementary data are available at *JXB* online.

Fig. S1. Physiological parameters measured.

Fig. S2. Accumulation pattern and fragmentation profile of the only signal over accumulated in *F. mosseae*-colonized tomato plants in the absence of herbivory.

Fig. S3. Classification of the major metabolic pathways according to *S. lycopersicum* KEGG and internal databases.

Fig. S4. Boxplots of selected metabolites with a primed accumulation pattern in response to local herbivory.

Table S1. Dataset containing metabolic profiles from tomato leaves.

Table S2. Dataset containing 200 signals with significantly different accumulation in at least one of the different treatments.

Table S3. Dataset containing 107 signals putatively identified by m/z and/ or fragmentation spectrum.

erab171_suppl_Supplementary_Figures_S1-S4

erab171_suppl_Supplementary_Tables_S1-S3

## Data Availability

All data supporting the findings of this study are available within the paper and within its supplementary materials published online.
