# Supplementary material for: Mycorrhizal symbiosis primes the accumulation of antiherbivore compounds and enhances herbivore mortality in tomato
Source: J Exp Bot. 2021 Apr 22;72(13):5038–50. doi: 10.1093/jxb/erab171 (PMC8219033; doi:10.1093/jxb/erab171)
Supplement: erab171_suppl_Supplementary_Figures_S1-S4 [file erab171_suppl_supplementary_figures_s1-s4.pdf]

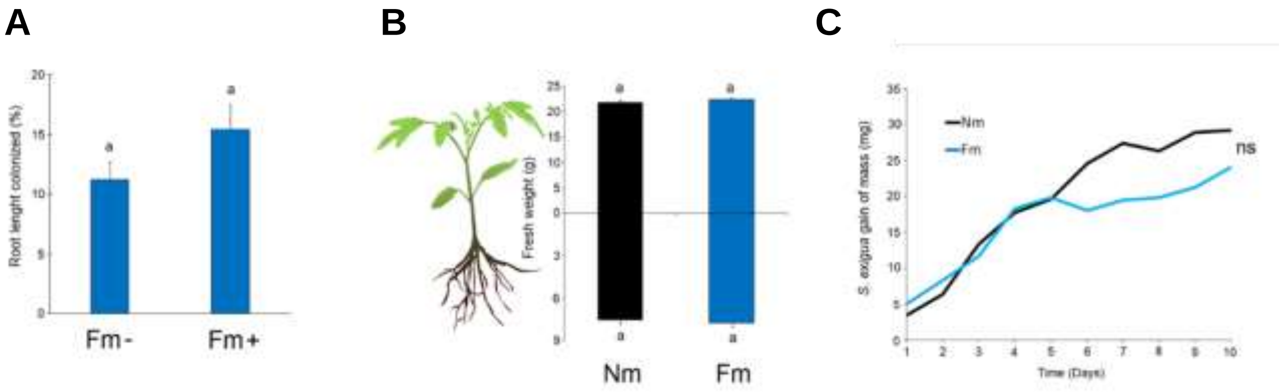

Fig. S1. Physiological parameters measured. (A) Percentage of root length colonized by *F. mosseae* in 8 weeks-old tomato plants grown in absence of stress (Fm-) or subjected to 2 weeks herbivory by *S. exigu*a (Fm+). (n=10). **(B)** Shoot and root fresh weight of 8 weeks-old of non-mycorrhizal (Nm, black bars) or AMF *F. mosseae* colonized tomato plants (Fm, blue bars) (n=10). **(C)** Weight gain of *Spodoptera exigua* larvae fed on six weeks old tomato plants colonized by AMF *F. mosseae* (Fm, blue line) or in absence of symbiosis (Nm, black line). Larvae biomass was counted every day until day 10. Only biomass of survivors was recorded. (n=10). Means not sharing a letter in common differ significantly according to the Fisher's LSD post hoc test ($P < 0.05$).

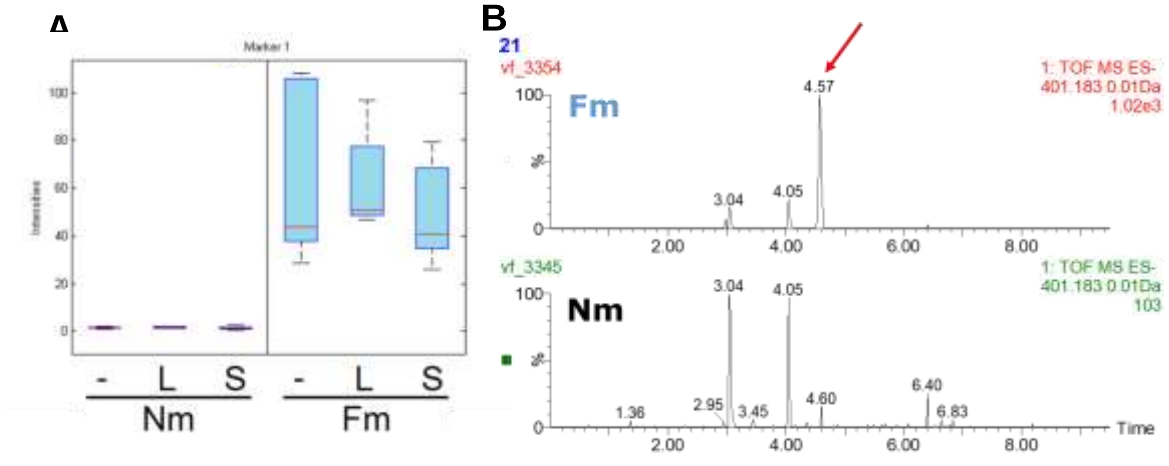

Fig. S2. Accumulation pattern and fragmentation profile of the only signal overaccumulated in *F. mosseae* colonized tomato plants in the absence of herbivory. (A) Boxplot representation of the putative metabolite with an exact m/z 402.190. **(B)** ESI-chromatogram of filtered ion mass 401.183, observing the peak at 4.57 min only in Fm plants. Nm, non mycorrhizal plant; Fm *F. mosseae* colonized plant, (-) no herbivory, (L) local –wounded- tissue, (S) systemic, unwounded tissue.

**Phenolic alcohols and derivatives**
Caffeine metabolism - Solanum lycopersicum (tomato)
Carotenoid biosynthesis - Solanum lycopersicum (tomato)
Lignans - Solanum lycopersicum (tomato)
Phenylpropanoid biosynthesis - Solanum lycopersicum (tomato)
Stilbenoid; diarylheptanoid and gingerol biosynthesis - Solanum lycopersicum (tomato)
**Terpenoid metabolism**
Diterpenoid biosynthesis - Solanum lycopersicum (tomato)
Limonene and pinene degradation - Solanum lycopersicum (tomato)
Monoterpenoid biosynthesis - Solanum lycopersicum (tomato)
Sesquiterpenoid and triterpenoid biosynthesis - Solanum lycopersicum (tomato)
Terpenoid backbone biosynthesis - Solanum lycopersicum (tomato)
Ubiquinone and other terpenoid-quinone biosynthesis - Solanum lycopersicum (tomato)
**Sugar metabolism**
Amino sugar and nucleotide sugar metabolism - Solanum lycopersicum (tomato)
Fructose and mannose metabolism - Solanum lycopersicum (tomato)
Galactose metabolism - Solanum lycopersicum (tomato)
Glycolysis / Gluconeogenesis - Solanum lycopersicum (tomato)
Pentose and glucuronate interconversions - Solanum lycopersicum (tomato)
**Flavonoid metabolism**
Anthocyanin biosynthesis - Solanum lycopersicum (tomato)
Flavone and flavonol biosynthesis - Solanum lycopersicum (tomato)
Flavonoid biosynthesis - Solanum lycopersicum (tomato)
**Amino acid metabolism**
Alanine; aspartate and glutamate metabolism - Solanum lycopersicum (tomato)
Aminoacyl-tRNA biosynthesis - Solanum lycopersicum (tomato)
Arginine and proline metabolism - Solanum lycopersicum (tomato)
beta-Alanine metabolism - Solanum lycopersicum (tomato)
Biosynthesis of amino acids - Solanum lycopersicum (tomato)
Cyanoamino acid metabolism - Solanum lycopersicum (tomato)
Cysteine and methionine metabolism - Solanum lycopersicum (tomato)
Glycine; serine and threonine metabolism - Solanum lycopersicum (tomato)
Histidine metabolism - Solanum lycopersicum (tomato)
Lysine degradation - Solanum lycopersicum (tomato)
Phenylalanine metabolism - Solanum lycopersicum (tomato)
Phenylalanine; tyrosine and tryptophan biosynthesis - Solanum lycopersicum (tomato)
Tropane; piperidine and pyridine alkaloid biosynthesis - Solanum lycopersicum (tomato)
Tryptophan metabolism - Solanum lycopersicum (tomato)
Tyrosine metabolism - Solanum lycopersicum (tomato)
Valine; leucine and isoleucine biosynthesis - Solanum lycopersicum (tomato)
Valine; leucine and isoleucine degradation - Solanum lycopersicum (tomato)

**Alkaloids**
Isoquinoline alkaloid biosynthesis - Solanum lycopersicum (tomato)
Nicotinate and nicotinamide metabolism - Solanum lycopersicum (tomato)
Tropane; piperidine and pyridine alkaloid biosynthesis - Solanum lycopersicum (tomato)
**ABC transporters**
ABC transporters
**Nucleotide metabolism**
Purine metabolism - Solanum lycopersicum (tomato)
Pyrimidine metabolism - Solanum lycopersicum (tomato)
**Vitamins**
Biotin metabolism - Solanum lycopersicum (tomato)
One carbon pool by folate - Solanum lycopersicum (tomato)
Riboflavin metabolism - Solanum lycopersicum (tomato)
Vitamin B6 metabolism - Solanum lycopersicum (tomato)
**Fatty acids derivatives**
alpha-Linolenic acid metabolism - Solanum lycopersicum (tomato)
Arachidonic acid metabolism - Solanum lycopersicum (tomato)
Glycerophospholipid metabolism - Solanum lycopersicum (tomato)
**Others**
2-Oxocarboxylic acid metabolism - Solanum lycopersicum (tomato)
Aminoacyl-tRNA biosynthesis - Solanum lycopersicum (tomato)
C5-Branched dibasic acid metabolism - Solanum lycopersicum (tomato)
Degradation of aromatic compounds - Solanum lycopersicum (tomato)
Glucosinolate biosynthesis - Solanum lycopersicum (tomato)
Glutathione metabolism - Solanum lycopersicum (tomato)
Oxidative phosphorylation - Solanum lycopersicum (tomato)
Photosynthesis - Solanum lycopersicum (tomato)
Plant hormone signal transduction - Solanum lycopersicum (tomato)
Plant-pathogen interaction - Solanum lycopersicum (tomato)
Porphyrin and chlorophyll metabolism - Solanum lycopersicum (tomato)
Selenocompound metabolism - Solanum lycopersicum (tomato)
Steroid biosynthesis

Fig. S3. Classification of the major metabolic pathways according to *S. lycopersicum* Kegg and internal databases.

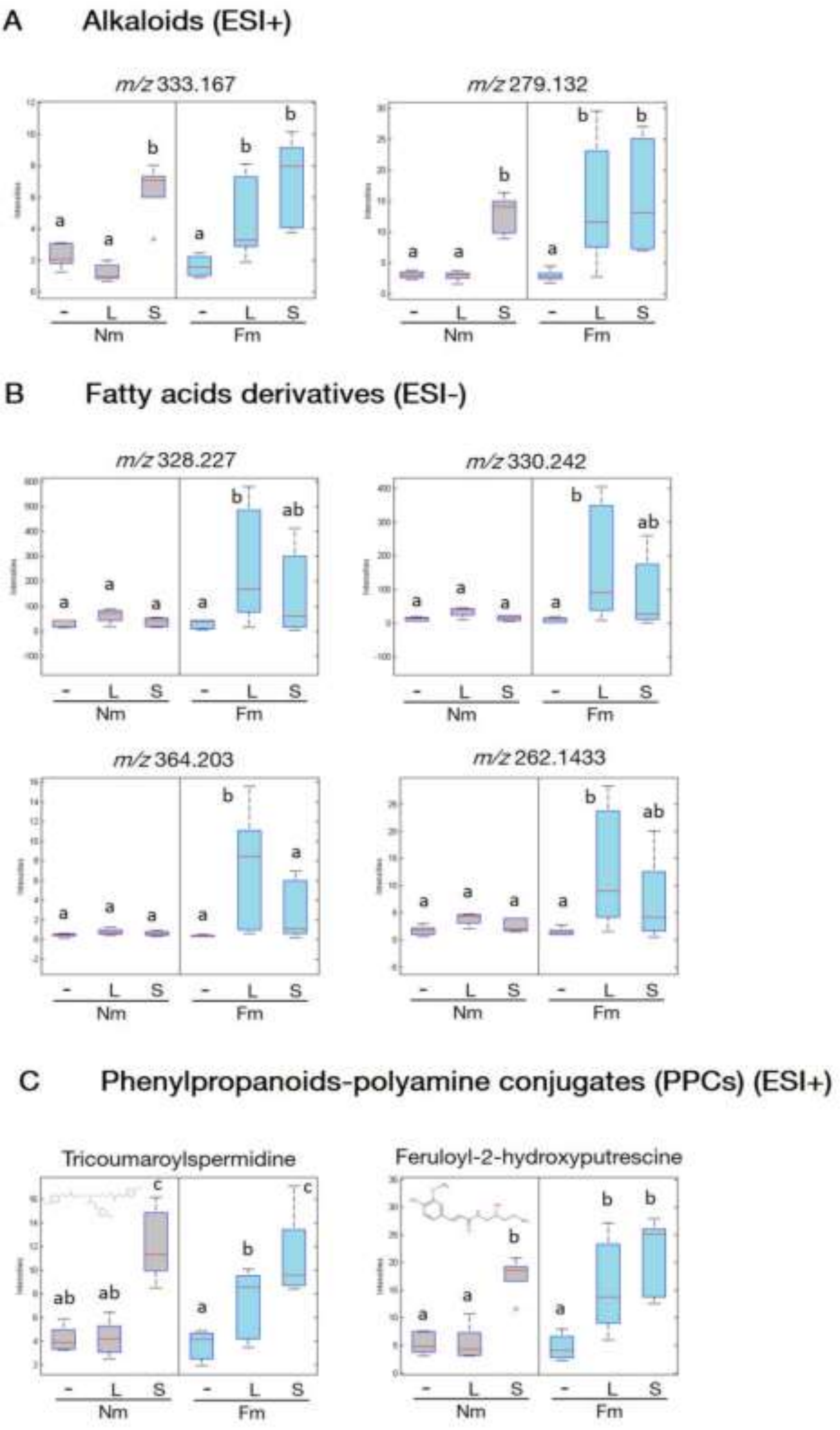

Fig. S4. Boxplots of selected metabolites with a primed accumulation pattern in response to local herbivory. Nm, non mycorrhizal plant; Fm *F. mosseae* colonized plant; (-) no herbivory; (L) local –wounded- tissue; (S) systemic, unwounded tissue.
